# Alcohol Use Disorder with and without Stimulant Use: Brain Morphometry and Its Associations with Cigarette Smoking, Cognition, and Inhibitory Control

**DOI:** 10.1371/journal.pone.0122505

**Published:** 2015-03-24

**Authors:** David L. Pennington, Timothy C. Durazzo, Thomas P. Schmidt, Christoph Abé, Anderson Mon, Dieter J. Meyerhoff

**Affiliations:** 1 Addiction Research Program, Veterans Administration Medical Center, San Francisco, California, United States of America; 2 Northern California Institute for Research and Education, San Francisco, California, United States of America; 3 Department of Radiology and Biomedical Imaging, University of California, San Francisco, California, United States of America; 4 Center for Imaging of Neurodegenerative Diseases, Veterans Administration Medical Center, San Francisco, California, United States of America; 5 Department of Clinical Neuroscience, Karolinska Institutet, Stockholm, Sweden; 6 School of Applied Sciences and Statistics, Koforidua Polytechnic, Ghana; University of Chicago, UNITED STATES

## Abstract

**Objective:**

Little is known about the effects of polysubstance use and cigarette smoking on brain morphometry. This study examined neocortical brain morphometric differences between abstinent polysubstance dependent and alcohol-only dependent treatment seekers (ALC) as well as light drinking controls (CON), the associations of cigarette smoking in these polysubstance users (PSU), and morphometric relationships to cognition and inhibitory control.

**Methods:**

All participants completed extensive neuropsychological assessments and 4 Tesla brain magnetic resonance imaging. PSU and ALC were abstinent for one month at the time of study. Parcellated morphological data (volume, surface area, thickness) were obtained with FreeSurfer methodology for the following bilateral components: dorso-prefrontal cortex (DPFC), anterior cingulate cortex (ACC), orbitofrontal cortex (OFC), and insula. Regional group differences were examined and structural data correlated with domains of cognition and inhibitory control.

**Results:**

PSU had significantly smaller left OFC volume and surface area and trends to smaller right DPFC volume and surface area compared to CON; PSU did not differ significantly from ALC on these measures. PSU, however, had significantly thinner right ACC than ALC. Smoking PSU had significantly larger right OFC surface area than non-smoking PSU. No significant relationships between morphometry and quantity/frequency of substance use, alcohol use, or age of onset of heavy drinking were observed. PSU exhibited distinct relationships between brain structure and processing speed, cognitive efficiency, working memory and inhibitory control that were not observed in ALC or CON.

**Conclusion:**

Polysubstance users have unique morphometric abnormalities and structure-function relationships when compared to individuals dependent only on alcohol and light drinking controls. Chronic cigarette smoking is associated with structural brain irregularities in polysubstance users. Further elucidation of these distinctive characteristics could help inform the development of targeted and thus potentially more effective treatments in this large but understudied population.

## Introduction

Magnetic resonance imaging methods can be used to identify unique structural differences between substance dependent populations as well as behaviorally significant neurocognitive correlates. Almost all neuroimaging studies performed in substance using populations to date have focused on persons ostensibly using single substances, either predominantly alcohol, cocaine, methamphetamine, opiates, or cannabis (e.g., [1, 2]). However, a large proportion of treatment seekers today use multiple substances concurrently, i.e., they are chronic polysubstance users (PSU) [3–5]. Specifying differences in brain morphology and cognitive performance between treatment-seeking PSU and monosubstance users may better inform personalized treatment of this understudied population [6, 7].

In previous magnetic resonance imaging based studies, we found that alcohol dependent treatment seekers with concurrent cocaine dependence had greater gross structural abnormalities (i.e., lobar tissue volumes) than cocaine dependent individuals without alcohol dependence [8, 9]. This was consistent with a study in abstinent polysubstance users (cocaine, alcohol, heroin, marijuana) that reported prefrontal cortical volume loss compared to non-drug using healthy controls (CON) [10]. Primary cocaine dependence has also been associated with smaller volumes and lower tissue density in prefrontal cortex (for reviews see [11, 12]). Further, these structural abnormalities may affect an individual’s ability to control reward-related behavior including the ability to achieve and maintain abstinence (see e.g., [13–15]). Therefore, identifying and characterizing behavioral correlates of such potentially distinct morphometric abnormalities in PSU may inform more efficient substance use treatment [16, 17].

Chronic cigarette smoking is almost ubiquitous among substance users [3–5, 18–20], and the cohorts in the above studies included both smoking and non-smoking individuals. The degree to which smoking mediates or moderates brain structure in PSU has not been examined although it may have implications for optimized approaches to effective substance use treatment. Cigarette smoking has been linked to widespread gray matter volume and neurocognitive abnormalities in non-clinical cohorts and those with alcohol use disorders (for review see [21]). Magnetic resonance imaging of adult smokers showed smaller volumes and/or lower gray matter densities throughout the cortical and subcortical brain, including the dorsal prefrontal cortex (DPFC), anterior cingulate cortex (ACC) and posterior cingulate cortex, as well as thinner orbitofrontal cortex (OFC) [22–27]. Greater morphological abnormalities in some brain regions correlated with greater pack years or severity of nicotine dependence [22–25]. Similarly, smoking exacerbates whole brain gray matter volume loss in both treatment-naïve heavy drinkers [28] and in 1-week-abstinent treatment-seeking alcohol dependent individuals [29] (although not replicated in a larger study when accounting for age effects [30]); smoking is also associated with thinner cortex as well as with greater age-related volume loss in the brain reward system of 1-week-abstinent alcohol-dependent treatment seekers (ALC) [31, 32]. Whole-lobe volume reductions in ALC largely normalized within one month of abstinence [33, 34], and recovery of frontal and total cortical gray matter as well as hippocampal volume [35, 36] blood flow [37], and neurocognition [38, 39] during abstinence from alcohol have been shown to be negatively impacted by chronic smoking. The foregoing abnormalities affect brain regions that are critically involved in inhibitory control, executive function, and reward processing, and neurobiological and neurocognitive abnormalities during abstinence may therefore underlie substance use behavior and ability to maintain abstinence [40–43]. As such, these functions constitute valuable targets for increasing the efficacy of treatment for alcohol/substance use disorders [44, 45].

Based on the brain morphometric literature in mono-substance using populations and cigarette smoking controls, we sought to determine if there are lasting morphometric abnormalities in one-month-abstinent PSU and the degree to which they relate to cigarette smoking, risk-taking, decision making, and other cognitive domains. We were specifically interested in measuring the structural integrity of neocortical brain regions that are critical for the development and maintenance of addictive disorders [46, 47], namely the DPFC, ACC, OFC, and insula. Specifically, we posited that one-month abstinent PSU exhibit abnormal morphometry in these neocortical regions, namely smaller cortical volumes, surface areas and thinner cortices than CON and—more informatively—compared to an age- and abstinence duration-matched cohort of ALC. We further hypothesized that smoking PSU have smaller cortical volumes, surface areas and thinner cortices in these frontal regions than non-smoking PSU. Finally, we aimed to explore relationships of regional morphometric abnormalities in PSU to specific aspects of cognition relevant to substance abuse.

## Methods

### Participants

All participants provided written informed consent prior to study according to the Declaration of Helsinki and underwent procedures approved by the Committee on Human Research, the Institutional Review Board of record for the University of California, San Francisco and the San Francisco VA Medical Center. **Table 1** shows group demographics and relevant substance use characteristics. Thirty-one treatment seeking PSU (21 smokers, 10 non-smokers) and 38 ALC (26 smokers, 12 non-smokers) were recruited from substance abuse treatment programs of the VA and Kaiser Permanente. Sixty-four CON (33 smokers, 31 non-smokers) with no history of biomedical and/or psychiatric conditions known to influence study measures were recruited from the local community. All ALC and PSU participants met DSM-IV criteria for alcohol dependence. In addition, PSU participants met DSM-IV criteria for dependence on at least one psychostimulant (29 cocaine and 9 amphetamine), with or without opioid dependence (n = 4) and cannabis use (n = 8). Specifically, 22 PSU met criteria for cocaine dependence; 2/22 also met opioid dependence and 3/22 used cannabis. Seven other PSU met criteria for both cocaine and amphetamine dependence; 1/7 also met opioid dependence and 3/7 used cannabis. Two other PSU met criteria for amphetamine dependence and 1/2 was also dependent on opioids. At the time of study ALC and PSU were abstinent from alcohol and other substances, except nicotine, for approximately one month. Participants were excluded for neurologic or psychiatric disorders known to affect neurobiology or neurocognition, such as HIV infection. Hepatitis C, type-2 diabetes, hypertension, and unipolar mood disorders were permitted given their high prevalence in alcohol/substance use disorders [4, 48–50]. Further inclusion and exclusion criteria are fully detailed elsewhere [51].

**Table 1:** Demographics, laboratory and substance consumption variables for ALC, PSU and CON (mean ± standard deviation).

				p	p	p
Variable	PSU	ALC	CON	PSU-ALC	PSU-CON	ALC-CON
**n**	31	38	64	-	-	-
**Age [years]**	46.8 ± 9.8	50.6 ± 8.8	46.5 ± 10.4	NS	NS	0.035
**Education [years]**	12.7 ± 1.3	13.4 ± 1.6	15.6 ± 2.7	NS	0.000	0.000
**AMNART**	106.6 ± 8.8	113.7 ± 8.8	118.0 ± 6.5	0.002	0.000	0.015
**Onset heavy drinking** ^**a**^ **[age]**	21.3 ± 8.1	24.1 ± 7.7	-	NS	-	-
**Months heavy drinking**	236.2 ± 131.7	257.2 ± 104.1	-	NS	-	-
**Sober days (alcohol and any drug)**	27.1 ± 10.3	33.1 ± 8.0	-	0.008	-	-
**1 year avg. alcohol [Drinks/month]**	297.2 ± 322.1	396.4 ± 254.7	19.7 ± 23.3	NS	0.002	0.000
**Life time avg. alcohol [Drinks/month]**	248.9 ± 255.4	210.9 ± 107.7	11.0 ± 15.8	NS	0.000	0.000
**Life time years drinking**	31.5 ± 10.0	33.9 ± 8.4	27.6 ± 12.5	NS	NS	0.032
**1 year avg. cocaine**	53.5 ± 37.6					
**[g/month] n (%)**	19 (61%)	-	-	-	-	-
**Life time avg. cocaine**	49.5 ± 39.2					
**[g/month] n (%)**	19 (61%)	-	-	-	-	-
**1 year avg.**						
**amphetamine**	11.3 ± 9.3					
**[g/month] n (%)**	7 (23%)	-	-	-	-	-
**Life time avg.**						
**amphetamine**	10.5 ± 9.8					
**[g/month] n (%)**	7 (23%)	-	-	-	-	-
**1 year avg. cannabis**	28.4 ± 28.5					
**[g/month] n (%)**	7 (26%)	-	-	-	-	-
**Life time avg.**						
**cannabis**	42.1 ± 24.6					
**[g/month] n (%)**	7 (26%)	-	-	-	-	-
**Smoker n (%)**	21 (68%)	26 (68%)	33 (52%)	-	-	-
**FTND total score**	4.2 ± 1.7	4.8 ± 1.6	4.8 ± 1.4	NS	NS	NS
**FTND cigarettes/day**	11.1 ± 7.2	17.5 ± 7.4	18.7 ± 6.6	0.004	0.000	NS
**FTND total lifetime years smoking**	22.0 ± 12.2	29.3 ± 8.3	26.8 ± 12.1	0.019	NS	NS
**BMI**	27.1 ± 6.7	26.5 ± 4.3	26.3 ± 3.6	NS	NS	NS
**BDI**	11.4 ± 8.1	13.3 ± 8.8	3.9 ± 3.8	NS	0.000	0.000
**STAI y2**	42.8 ± 10.2	46.3 ± 11.3	29.2 ± 6.8	NS	0.000	0.000
**STAXI-2**	51.7 ± 2.1	50.6 ± 2.4	42.0 ± 3.9	NS	0.030	NS
**HEP-C n (%)**	3 (10%)	2 (5%)	0 (0%)	NS	-	-
**Substance-Induced Mood Disorder n (%)**	3 (10%)	3 (8%)	0 (0%)	NS	-	-
**Mood D/O n (%)**	5 (16%)	9 (24%)	0	NS	-	-

NS = not significant (p>0.05);

^a^ heavy drinking defined as >100 alcoholic drinks/mo in men and >80 alcoholic drinks/mo in women;

AMNART, American National Adult Reading Test; avg., average; g, gram; FTND, Fagerstrom Tolerance Test for Nicotine Dependence; BMI, Body Mass Index; BDI, Beck Depression Inventory; STAI, State-Trait Anxiety Inventory, Y-2; STAXI, State-Trait Anger Expression Inventory-2; HEP-C, Hepatitis C; substance use for cocaine, methamphetamine and cannabis was not collected for all participants; opioid use was not collected for any participant.

### Psychiatric/behavioral assessment

All participants completed the Structured Clinical Interview for DSM-IV Axis I Disorder Patient Edition, Version 2.0 [52]. Within one day of the magnetic resonance study, all participants filled out questionnaires that assessed depression (Beck Depression Inventory [53]) and anxiety symptoms (State-Trait Anxiety Inventory, Y-2 [trait anxiety] [54]), as well as anger expression (State-Trait Anger Expression Inventory-2 [55]). Alcohol consumption in all participants was assessed with the Lifetime Drinking History semi-structured interview [56–58], which yielded estimates of the average number of standard alcoholic drinks (containing 13.6 g of ethanol) consumed per month, one year before enrollment and over lifetime.

For PSU, lifetime substance use history was assessed with an in-house interview questionnaire based on the Addiction Severity Index [59], NIDA Addictive Drug Survey [60], lifetime drinking history [56–58], and Axis I disorders Patient Edition, Version 2.0 (SCID-I/P [52]). This instrument gathers information relevant to drug use for each substance for which a participant has a current or past substance use diagnosis; this includes date of last use, and frequency and quantity of use. It also includes conversion of money spent per day to one metric, using catchment area-specific conversion norms. Thus, monthly averages for grams of cocaine, methamphetamine and/or cannabis over 1 year prior to enrolment and over lifetime were estimated. As this substance use assessment instrument was developed during the course of the study, not all participants provided the necessary data in the exact same quantifiable format. Level of nicotine dependence was assessed via the Fagerstrom Tolerance Test for Nicotine Dependence[61, 62], and total numbers of years of smoking and average number of daily cigarettes currently smoked were recorded.

### Neurocognitive assessment

Participants completed a comprehensive battery, which evaluated the adverse consequences of alcohol/substance use disorders [63–66] and chronic cigarette smoking [67, 68] on neurocognition. The neurocognitive domains evaluated and their constituent measures were as follows (for corresponding references see [69]: Executive functions: Short Categories Test, color-word portion of the Stroop Test, Trail Making Test B, Wechsler Adult Intelligence Scale 3rd Edition (WAIS-III) Similarities, Wisconsin Card Sorting Test-64: Computer Version 2-Research Edition non-perseverative errors, perseverative errors, and perseverative responses. General intelligence: Ward-7 Full Scale IQ (based on WAIS-III Arithmetic, Block Design, Digit Span, Digit Symbol, Information, Picture Completion, and Similarities subtests). Learning and memory: Auditory-verbal: California Verbal Learning Test-II, Immediate Recall trials 1 to 5 (learning), average of Short and Long Delay Free Recall (memory). Visuospatial: Brief Visuospatial Memory Test-Revised, Total Recall (learning) and Delayed Recall (memory). Processing speed: WAIS-III Digit Symbol, Stroop Color & Word, WAIS-III Symbol Search, Trail Making Test A. Visuoperceptual Skills: WAIS-III Block Design; Luria-Nebraska Item 99. Working memory: WAIS-III Arithmetic, WAIS-III Digit Span. Cognitive efficiency: This domain consisted of all tests that were timed, or in which the time to complete the task influenced the score achieved and was calculated by averaging the individual z-scores of those measures. Timed tests included the Luria-Nebraska Item 99, Stroop word, color, and color-word tests, Trail Making Tests A and B and WAIS-III Arithmetic, Block Design, Digit Symbol, Picture Completion, and Symbol Search. Higher scores on these measures reflect better speed and accuracy on principally nonverbal tasks. The cognitive efficiency domain approximates the concept of cognitive efficiency described previously [70–72]. Premorbid verbal intelligence was estimated with the American National Adult Reading Test.

Raw scores for each cognitive measure were converted to z-scores based on the performance of CON. Domain scores with multiple measures represent the average of the individual z-scores of the constituent measures of the domain. A global neurocognitive functioning score was calculated from the arithmetic mean of z-scores for all individual domains (excluding fine motor skills).

### Tasks of inhibitory control (risk-taking, decision-making, and impulsivity)

Participants completed the Balloon Analogue Risk Task (BART [73]), a measure of risk taking, the Iowa Gambling Task (IGT [74–76]), a measure of decision making, and the Barratt Impulsivity Scale-11 (BIS-11[77]), a self-report questionnaire that assesses impulsivity.

### Magnetic resonance image acquisition and processing

Magnetic resonance imaging data were acquired on a 4 Tesla Bruker MedSpec system with a Siemens Trio console (Siemens, Erlangen, Germany) using an 8-channel transmit-receive head coil. A Magnetization Prepared Rapid Gradient (TR/TE/TI = 2300/3/950 ms, 7° flip angle, 1.0 x 1.0 x 1.0 mm^3^ resolution) and a turbo spin-echo (TR/TE = 8400/70 ms, 150° flip angle, 0.9 x 0.9 x 3 mm^3^ resolution) sequences were used to acquire 3-D sagittal T1-weighted and 2D axial T2-weighted anatomical images, respectively.

The publicly available FreeSurfer (v5.1) volumetric segmentation and cortical surface reconstruction methods were used to obtain regional measures of cortical volume, surface area, and thickness. These measures have unique properties: While cortical surface area and volume are thought to be under genetic control (i.e., highly heritable), cortical thickness, an indicator of the integrity of cytoarchitecture in the cortex [78], is thought to be more strongly modulated by environmental factors [79, 80]. Since volume is the product of these metrics, their separate evaluations potentially enhances both accuracy and sensitivity for detecting group differences in cortical morphometry. FreeSurfer processing includes motion correction and averaging of volumetric T1 weighted images, removal of non-brain tissue using a hybrid watershed/surface deformation procedure, automated Talairach transformation, segmentation of the subcortical white matter and deep gray matter volumetric structures intensity, normalization, tessellation of the gray matter-white matter boundary, automated topology correction, and surface deformation. Spatial normalization to a template cortical surface allowed automatic parcellation of the cortical surfaces into 34 anatomical regions of interest (ROI) per hemisphere, and thickness measures were obtained for all 34 bilateral ROIs (for technical details and reliability see [81, 82]).

**Fig 1:**
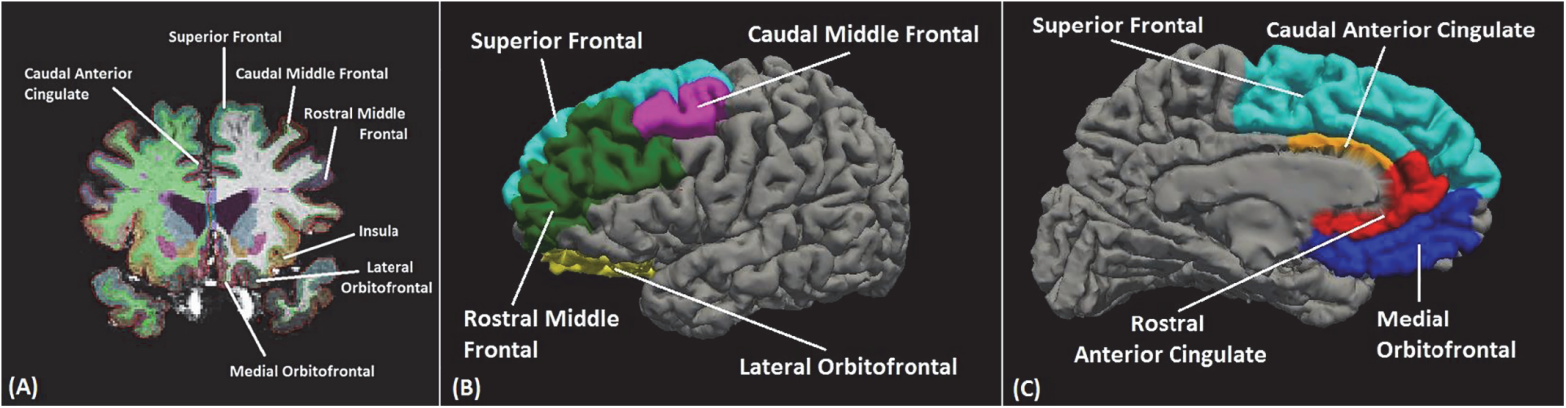
Sample FreeSurfer segmented image. Coronal (A), lateral (B) and medial (C) image showing typical automated cortical segmentation results from FreeSurfer. Different brain regions are indicated by different colors. Visible sub regions used in this study are labeled.

For this study, the FreeSurfer labeled ROIs were: ACC—rostral and caudal; DPFC—rostral and caudal middle frontal and superior frontal gyri; OFC—medial and lateral; and insula—standard FreeSurfer label (see **Fig. 1**). For the ACC, DPFC and OFC composite regions, an average thickness was calculated from the individual anatomical labels weighted by their surface area contribution that constituted each region. FreeSurfer also provides a measure of intracranial volume, estimated based on the Talairach transform [83].

### Statistical analyses

Separate univariate analyses of covariance examined group (PSU vs. ALC vs. CON) differences of three morphometric measures (volume, surface area, thickness) among four bilateral cortical ROIs (ACC, DPFC, OFC, insula). Univariate analysis of covariance was also used to examine differences between smoking and non-smoking PSU. Each of these analyses was controlled for age and intracranial volume. Follow up pairwise comparisons between PSU and ALC also included duration of abstinence (i.e., days sober) as a covariate. We left covariates in the final model only when they predicted significant group differences. We accounted for the multiplicity of morphometric measures in each ROI by correcting alpha levels via a modified Bonferroni procedure [84]. This approach yields adjusted alpha levels for each ROI separately using the number of morphometric measures under investigation (three in two hemispheres) and their average inter-correlation coefficients over all ROIs (DPFC: r = 0.496., ACC: r = 0.355, OFC: r = 0.437, insula: r = 0.418); the corresponding adjusted alpha levels were 0.020 for DPFC, 0.016 for ACC, 0.018 for OFC, and 0.017 for insula. Given the large amount of analyses and to reduce type I error, the investigation of smoking effects were limited to PSU. Effect sizes were calculated via Cohen’s d [85]. We correlated ROI-specific morphometric measures with neurocognitive z-scores using Spearman’s rho. Since these were exploratory hypotheses, we chose a less restrictive alpha level of 0.025 (half of p = .05) to adjust for multiple comparisons of these non-independent measures. Nevertheless these correlational analyses should be considered exploratory. All analyses used SPSS v21 software [86].

## Results

### Participant Characterization (Table 1)

One hundred and thirty-three male volunteers participated in study procedures. Seventeen of the 31 PSU participants were African American (55%), nine of [Western] European descent (29%), three Latino (10%), and one each Native American and Polynesian/Pacific Islander (3%). The group of 38 ALC patients was comprised of 25 [Western] European descent (65%), seven African Americans (18%), four Latinos (11%), and one each Native American and Other (3%). Of the 64 CON participants, 43 were of [Western] European descent (67%), nine Latino (14%), six African American (9%), five Asian American (8%), and one Pacific Islander (2%). Ethnic distribution was different between the three groups (χ2 = 45.76, p<0.001). However, in follow-up univariate analyses of covariance ethnicity was not a significant predictor and, thus, was not included in the final models. ALC were older than CON, but mean PSU age did not differ from either group. All three groups differed on premorbid verbal intelligence scores (PSU<ALC<CON). PSU did not differ from ALC on years of education, but both groups had less education than CON. ALC had been abstinent for 6 more days than PSU at the time of assessment, but they were otherwise matched on measures of drinking severity (average monthly drinks consumed in the past year and over lifetime, total lifetime drinking years). Although level of nicotine dependence was similar across groups, PSU smoked fewer cigarettes per day than both ALC and CON and had fewer smoking years than ALC. Both PSU and ALC had significantly higher depressive and anxiety symptoms than CON. PSU did not differ from ALC, but they had higher anger expression scores than CON. The PSU group did not differ from the ALC group in the proportions of individuals with comorbid mood disorder (<25%) or hepatitis-C (≤10%). Covarying for hepatitis-C and excluding hepatitis-C patients from analyses did not alter our results significantly. By design, all PSU and ALC were attending outpatient addiction treatment, 5/31 (16%) PSU and 12/38 (32%) ALC were also receiving pharmacotherapy for alcohol treatment, and 9/31 (29%) PSU and 12/38 (32%) ALC were taking other psychiatric medication (anti-depressant/anxiolytic). PSU and ALC did not significantly differ in proportion receiving alcohol or other psychiatric pharmacotherapy (χ2 = 2.19, p<0.138 and χ2 = 0.052, p<0.819, respectively).

### PSU, ALC, and CON comparison of ROI morphometry

Univariate analyses of covariance comparing PSU, ALC, and CON were significant for left OFC volume (F(2,123) = 5.07, p = 0.008) and right ACC thickness (F(2,123) = 4.48, p = 0.013) and tended to be different for left OFC surface area (F(2,123) = 3.12, p = 0.048). **Table 2** shows mean morphometric measurements by bilateral ROI and group, pairwise statistics, and effect sizes for two-group comparisons. Follow-up pairwise comparisons showed that PSU had smaller volume and surface area of the left OFC than CON (p≤0.015), but they did not differ from ALC on these measures. PSU also had thinner right ACC cortex than ALC, but not CON (i.e., ALC had thicker right ACC cortex than both PSU and CON, both p≤0.009).

**Table 2:** Morphometric differences between PSU-ALC, PSU-CON, and ALC-CON in DPFC, ACC, OFC and Insula.

ROI	Measure	Side	PSU Means (SD)	ALC Means (SD)	CON Mean (SD)	PSU-ALC	PSU-CON	ALC-CON
						p (ES)	p (ES)	p (ES)
		LEFT	43719.2 ± 6107.0	42526.1 ± 5260.5	45187.6 ± 4333.5	NS	NS	0.099^$^ (-.55)
	VOLUME	RIGHT	43427.0 ± 5699.2	43327.8 ± 5321.6	45317.0 ± 4715.8	NS	0.080^$^ (-.36)	NS
		LEFT	15480.8 ± 1957.4	15492.2 ± 1780.2	16064.2 ± 1507.7	NS	NS	NS
DPFC	S-AREA	RIGHT	15682.9 ± 1884.2	15855.8 ± 1790.0	16335.7 ± 1597.3	NS	0.077^$^ (-.37)	NS
		LEFT	2.41 ± 0.08	2.37 ± 0.09	2.40 ± 0.09	0.059^$^ (+.47)	NS	NS
	THICKNESS	RIGHT	2.39 ± 0.08	2.37 ± 0.09	2.37 ± 0.09	NS	NS	NS
		LEFT	4621.7 ± 1055.5	4633.6 ± 877.9	4733.1 ± 828.3	NS	NS	NS
	VOLUME	RIGHT	4198.2 ± 974.7	4157.1 ± 869.4	4152.3 ± 916.5	NS	NS	NS
		LEFT	1628.7 ± 333.0	1650.5 ± 286.6	1672.5 ± 240.4	NS	NS	NS
ACC	S-AREA	RIGHT	1609.6 ± 324.1	1547.6 ± 286.8	1619.6 ± 292.6	NS	NS	NS
		LEFT	2.53 ± 0.13	2.53 ± 0.18	2.54 ± 0.19	NS	NS	NS
	THICKNESS	RIGHT	2.26 ± 0.16	2.36 ± 0.17	2.24 ± 0.17	**0.009** ^**$$**^ **(-.60)**	NS	**0.005** ^**$$**^ **(+.69)**
		LEFT	11675.6 ± 1468.4	11739.9 ± 1358.7	12475.4 ± 1223.1	NS	**0.003** ^**$$**^ **(-.59)**	0.082^$^ (-.57)
	VOLUME	RIGHT	11648.9 ± 1577.1	11356.0 ± 1395.9	11693.7 ± 1065.3	NS	NS	NS
		LEFT	4549.3 ± 533.3	4628.9 ± 514.9	4791.1 ± 407.4	NS	**0.015** ^**$$**^ **(-.50)**	NS
OFC	S-AREA	RIGHT	4660.3 ± 617.2	4530.7 ± 529.4	4666.3 ± 405.9	NS	NS	NS
		LEFT	2.30 ± 0.12	2.26 ± 0.12	2.32 ± 0.12	NS	NS	0.077^$^ (-.50)
	THICKNESS	RIGHT	2.18 ± 0.12	2.19 ± 0.10	2.17 ± 0.10	NS	NS	NS
		LEFT	6790.0 ± 812.6	6750.5 ± 1036.8	7058.3 ± 658.8	NS	NS	NS
	VOLUME	RIGHT	7009.9 ± 966.4	7010.8 ± 974.5	7263.7 ± 779.4	NS	NS	NS
		LEFT	2326.4 ± 241.8	2356.7 ± 355.2	2405.0 ± 222.6	NS	NS	NS
INSULA	S-AREA	RIGHT	2399.9 ± 350.5	2433.9 ± 318.8	2453.8 ± 257.7	NS	NS	NS
		LEFT	2.81 ± 0.13	2.77 ± 0.18	2.84 ± 0.14	NS	NS	NS
	THICKNESS	RIGHT	2.83 ± 0.13	2.78 ± 0.18	2.86 ± 0.14	NS	NS	0.061^$^ (-.50)

^$$^ significant at adjusted alpha level (p<0.018 for OFC; p<0.016 for ACC);

^$^ trends (p<0.10); S-AREA, surface area;

NS, not significant; SD, standard deviation; ES, effect size; mean volume in mm^3^; mean surface area in mm^2^; mean thickness in mm.

Although univariate tests for group differences in the DPFC were not significant, planned follow-up comparisons showed trends (p≤0.10) to thicker left DPFC cortex in PSU than ALC (p = 0.059). Volume and surface area of the right DPFC tended to be smaller in PSU than CON (p≤0.08), but similar to the measures in ALC. Finally, ALC showed trends to smaller left DPFC and OFC volumes (both p≤0.099) as well as to thinner right insula and left OFC cortices (both p≤0.077) than CON.

None of our substance abuse or alcohol use measures including quantity, duration, or age of onset of heavy drinking was correlated significantly with our morphometric measures.

### Comparisons of smokers and non-smokers in PSU and ALC

**Table 3** shows mean morphometric measurements by bilateral ROI in smoking and non-smoking PSU, pairwise statistics, and effect sizes. Pairwise comparisons revealed that the 21 smoking PSU had a larger right OFC surface area than the 11 non-smoking PSU (p = 0.015). In addition, smoking vs. non-smoking PSU showed trends to larger right OFC volume, larger right DPFC surface area (both p<0.035), larger left ACC volume and surface area (both p<0.053) and thicker left ACC (p = 0.024). The effect sizes were moderate to strong, between 0.56 and 0.90 (see **Table 2**). By contrast, similar smoking effects were not observed in the larger 1-month-abstinent ALC group of this study. Therefore, when using smoking status as a covariate in additional follow-up analyses that compared PSU to ALC, smoking status was not a significant predictor of these cortical measures. However, the thicker left ACC in sPSU was related to greater smoking severity as reflected in a higher Fagerstrom total score (r = 0.559, p = 0.010).

**Table 3:** PSU morphometric smoking differences in DPFC, ACC, OFC and Insula.

Region	Measure	Side	p (ES)	Means (SD)	
				Smoking	Non-Smoking
		LEFT	NS	44171.3 ± 6686.7	42815.0 ± 4941.8
	VOLUME	RIGHT	NS	44162.0 ± 6448.9	41957.0 ± 3642.0
		LEFT	NS	15718.7 ± 2100.4	15005.1 ± 1629.2
DPFC	S-AREA	RIGHT	0.028^$^ (.58)	16013.4 ± 2036.5	15022.1 ± 1398.8
		LEFT	NS	2.40 ± 0.08	2.43 ± 0.08
	THICKNESS	RIGHT	NS	2.38 ± 0.09	2.40 ± 0.08
		LEFT	0.031^$^ (.64)	4838.1 ± 1044.3	4188.8 ± 987.4
	VOLUME	RIGHT	NS	4355.1 ± 1055.5	3884.4 ± 738.6
		LEFT	0.053^$^ (.56)	1688.7 ± 340.8	1508.9 ± 296.7
ACC	S-AREA	RIGHT	NS	1664.0 ± 351.9	1500.7 ± 239.6
		LEFT	0.024^$^ (.90)	2.57 ± 0.11	2.46 ± 0.13
	THICKNESS	RIGHT	NS	2.27 ± 0.15	2.25 ± 0.19
		LEFT	NS	11895.8 ± 1631.0	11235.2 ± 1004.2
	VOLUME	RIGHT	0.035^$^ (.74)	11994.6 ± 1679.7	10957.6 ± 1123.3
		LEFT	NS	4616.0 ± 576.2	4415.8 ± 431.2
OFC	S-AREA	RIGHT	**0.015** ^**$$**^ **(.90)**	4822.0 ± 640.4	4337.0 ± 432.7
		LEFT	NS	2.32 ± 0.14	2.27 ± 0.06
	THICKNESS	RIGHT	NS	2.18 ± 0.11	2.19 ± 0.13
		LEFT	NS	6920.4 ± 834.3	6485.78 ± 710.7
	VOLUME	RIGHT	NS	7008.1 ± 1094.4	7014.22 ± 625.7
		LEFT	NS	2355.3 ± 264.1	2258.89 ± 174.0
INSULA	S-AREA	RIGHT	NS	2396.1 ± 392.3	2408.67 ± 246.0
		LEFT	NS	2.83 ± 0.13	2.76 ± 0.12
	THICKNESS	RIGHT	NS	2.84 ± 0.13	2.80 ± 0.12

^$$^ significant at adjusted alpha level (p<0.018 for OFC);

^$^ trends (p<0.10); S-AREA, surface area;

NS, not significant; SD, standard deviation; mean volume in mm^3^; mean surface area in mm^2^; mean thickness in mm.

### Associations of morphometric measures with neurocognition (Table 4)

**Table 4** shows significant correlations (p<0.025 after correction for multiple comparisons) between measures of cortical morphometry and cognition within the three groups (i.e., PSU, ALC, and CON). Most correlations were observed in ALC: Smaller volumes and surface areas of the DPFC, OFC, and insula correlated with worse performance in many domains of neurocognition (i.e., cognitive efficiency, executive function, intelligence, visuospatial learning and memory, working memory), while smaller ACC volumes and surface areas correlated with worse working memory (all r>0.41). Within PSU, smaller DPFC volumes and surface areas as well as smaller left OFC volumes were correlated with worse global cognition and the following constituent domains: cognitive efficiency, executive function, intelligence, processing speed; in addition, smaller left ACC volume and surface area were related to worse performance in cognitive efficiency and processing speed (all r>0.47). In CON, very few such associations were significant (all r>0.35).

**Table 4:** Significant Spearman correlations (rho) between cortical morphometric measures and neurocognitive domains within PSU, (ALC), and [CON].

	MEASURE	SIDE	AV	Cog	Exec	Fine	Gen	Proc	VS	VS	VP	Wk	Global
			Mem	Eff	Fx	Mot	Intel	Sp	Learn	Mem	Skills	Mem	Cog
		LEFT		.57 (.41)	.56 (.44)		.59 (.49)	.64	(.48)			(.66)	.50 (.45)
	VOLUME	RIGHT		.64 (.44)	.51 (.50)		.57 (.43)	.68	(.55)	(.49)	.47 (.44)	(.57)	.50 (.50)
		LEFT		.56 (.40)	.56 (.41)		.57 (.49)	.63	(.42)			(.61)	.50
DPFC	S-AREA	RIGHT		.65 (.43)	.52 (.45)		.55 (.45)	.71	(.49)	(.43)		(.52)	.49 (.42)
		LEFT											
	THICKNESS	RIGHT											
		LEFT		.56	(.42)			.55				(.53)	
	VOLUME	RIGHT										(.49)	
		LEFT		.51				.53				(.43)	
ACC	S-AREA	RIGHT					(.45)	.49				(.49)	
		LEFT											
	THICKNESS	RIGHT			-.47								
		LEFT		.53	.48 (.48)	[.36]	.62 (.52)	.49	(.44)		.48 (.42)	(.52)	.49
	VOLUME	RIGHT			(.47)		(.46)				(.44)	(.51)	
		LEFT					(.45)					(.42)	
OFC	S-AREA	RIGHT		(.43)	(.44)		(.59)					(.51)	
		LEFT	.43		(.54)						(.43)		.50 (.43)
	THICKNESS	RIGHT											
		LEFT		(.53)	(.50)	(.57)	(.48)				(.46)	(.53)	(.43)
	VOLUME	RIGHT		(.44)	(.43)	(.45)							
		LEFT		(.48)		(.47)	(.45)					(.52)	
INSULA	S-AREA	RIGHT				[.38]		.53 [.38]					.50
		LEFT			(.42)			.48					
	THICKNESS	RIGHT											

All p<0.025; S-AREA, surface area; AV Mem, auditory-verbal memory; Cog Eff, cognitive efficiency; Exec Fx, executive function; Fine Mot, fine motor skills; Gen Intel, general intelligence; Proc Sp, processing speed; VS Learn, visuospatial learning; VS Mem, visuospatial memory; VP Skills, Visuoperceptual skills, Wk Mem, working memory; Cog, cognition.

### Associations of morphometric measures with BIS-11, BART, and IGT (Table 5)

**Table 5** depicts significant (p<0.025 after correction for multiple comparisons) correlations between cortical morphometry and BIS-11, BART, and IGT within the three groups. Significant weak-to-moderate correlations (r = 0.38–0.51) were observed, but without overlap between the groups. In PSU, thicker cortices in left DPFC were associated with lower BIS-11 Attention impulsivity; similarly in CON, thicker cortices in the right DPFC correlated with lower BIS-11 Total and Attention impulsivity. In ALC, smaller left insular volume related to lower BIS-11 Attention impulsivity; similarly in CON, smaller left insular surface area related to lower BIS-11 Total and Non-Planning impulsivity. However, thicker right insula cortices were associated with lower BIS-11 Non-Planning impulsivity in CON.

**Table 5:** Significant Spearman correlations (rho) between cortical morphometrics and measures of inhibitory control within PSU, (ALC), and [CON].

	MEASURE	SIDE	BIS	BIS Non-	BIS Total	BART Pumps	IGT Net Total
			Attention	Planning		Adjusted Avg.	t-score
		LEFT					
	VOLUME	RIGHT				.52	[-.42]
		LEFT				.60	[-.53]
DPFC	S-AREA	RIGHT				.52	[-.56]
		LEFT	-.47				
	THICKNESS	RIGHT	[-.43]		[-.42]		
		LEFT					[-.41]
	VOLUME	RIGHT				.62	
		LEFT					[-.55]
ACC	S-AREA	RIGHT				.62	
		LEFT					
	THICKNESS	RIGHT					[.45]
		LEFT				[.41]	
	VOLUME	RIGHT				[.40]	
		LEFT					
OFC	S-AREA	RIGHT					
		LEFT					
	THICKNESS	RIGHT					
		LEFT	(.51)				
	VOLUME	RIGHT					
		LEFT		[.40]	[.38]		
INSULA	S-AREA	RIGHT					
		LEFT					
	THICKNESS	RIGHT		[-.41]			

All p<0.025; S-AREA, surface area; BIS, Barratt Impulsivity Scale-11; BART, Balloon Analogue Risk Task; IGT, Iowa Gambling Task.

In PSU, higher BART Adjusted Average Pumps (commensurate with greater risk-taking) correlated significantly with larger volume in the right DPFC, larger surface area in bilateral DPFC, and larger volume and surface area in the right ACC (all p<0.02, all r = 0.52–0.62). There were no corresponding associations in ALC. In CON, higher BART Adjusted Average Pumps related to larger bilateral OFC volume (r = 0.40 and 0.41).

Structure-function relationships were not observed with IGT- in either PSU or ALC. In CON, however, lower IGT Net Total t-scores (commensurate with worse decision-making) related to larger bilateral DPFC surface areas, larger right DPFC volume, larger surface area and volume of the left ACC, as well as to thinner right ACC cortex (all p<0.02, all r>-0.40).

## Discussion

One-month-abstinent PSU dependent on both alcohol and at least one psychostimulant showed prefrontal morphometric differences compared to non-drug using, light drinking healthy controls. By contrast, such abnormalities were not observed in ALC matched on age, sex, education, smoking status, and abstinence duration to PSU and similar on duration and onset-age of heavy drinking. Specifically, PSU had significantly thinner right ACC than ALC, but were not different from CON on this measure. PSU also had significantly smaller left OFC volume and surface area than CON. The insula was largely spared from morphometric abnormalities in both abstinent substance dependent groups. Smoking status in PSU affected morphometric measures from the OFC, ACC, and DPFC; this smoking effect remained only significant for the OFC surface area after corrections for multiple comparisons. While greater smoking severity correlated with thicker ACC in PSU, it did not affect any of our measures of cognitive functioning. PSU also exhibited unique functional relationships to morphometry different from those in ALC or CON.

### Morphometric abnormalities in PSU

Our one-month-abstinent PSU had significantly thinner right ACC than ALC but were not different from CON on this measure. Conversely, ALC had significantly thicker right ACC compared to CON. Previous reports found no ACC thickness abnormalities in stimulant-dependent (most also dependent on alcohol) adults with recent use [87], approximately 53 days of abstinence [88], and in those with up to 1.5 years of abstinence [89]. Yet, Durazzo et al., [90] showed participants dependent only on alcohol had thinner right caudal ACC than controls at approximately 1 week of abstinence. Taken together, the results suggest polysubstance dependence (including alcohol) was not associated with ACC structural abnormalities in the first month (possibly up to 1.5 years) of abstinence from substance use, whereas alcohol dependence alone showed ACC dysmorphology during this same timeframe.

Significantly smaller left OFC volume in PSU compared to CON is generally consistent with studies of stimulant dependence in active users [91–93]. Reports also showed that cocaine dependence was associated with lower gray matter tissue density in medial OFC [94] and methamphetamine dependence was associated with reduced OFC volume [95] in participants with similar abstinence duration (~18–20 days) to those of the current study (~27 days). A study of polysubstance users (primarily cocaine dependent) with approximately 60 days of abstinence showed reduced medial OFC volume compared to controls [96]. The OFC abnormalities observed in PSU are likely associated with the collection of symptoms characteristic of polysubstance use rather than transient drug effects or premorbid disposition, in part because we did not observe any associations between substance consumption and morphological measures in PSU, and because morphometric OFC abnormalities are not typically observed in stimulant users with longer abstinence duration (>60 days) (for review see [12]).

Our findings of trends to smaller DPFC in PSU compared to CON were also similar to findings in actively using [97] and recently abstinent (~42 and 132 days, respectively) [8, 9] participants with both cocaine and alcohol use disorder and in recently (~18 days) abstinent participants with methamphetamine dependence [95]. DPFC abnormalities in crack-cocaine and alcohol dependent men are seen at up to 132 days of abstinence [9]. Thus, the trends for DPFC abnormalities of PSU in the current report likely reflect relatively long-lasting effects of drug use and/or premorbid disposition. However, due to the cross-sectional nature of our analyses, a definitive supposition cannot be made and the interpretations are made with caution.

Volume generally exhibits a linear relationship with surface area [98], so it is not surprising that we also observed significantly reduced left OFC and trends to reduced right DPFC surface area in PSU compared to CON. However, to our surprise, we found no morphometric abnormalities in the insula of PSU or ALC. Others found smaller insular volume compared to controls in actively using cocaine [91] and stimulant dependent individuals [93], as well as in abstinent (18–64 days) methamphetamine dependent [95, 99] participants. Conversely, thicker insula cortices were observed in a group of male stimulant dependent participants (many of whom also used alcohol/opiate/cannabis) with approximately 1.5 years of abstinence [89]. Given these conflicting results, the associations of polysubstance use on insula morphology is less clear.

### Smoking effects

In our modest sample of 1-month-abstinent PSU, smokers had significantly *larger* right OFC surface area than non-smokers, trends to *larger* right OFC volume, and trends to *larger* morphometric measures in left ACC. No significant smoking effects on any cortical measure were observed in our 1-month-abstinent ALC. By contrast, in a larger cohort of ALC abstinent for only one week, smokers had thinner cortices in the ACC, insula, and total frontal cortex than non-smokers [32], but they did not differ from non-smoking ALC on any regional cortical volume [34, 46]. In drug-free controls, smokers also had smaller volumes than non-smokers in the DPFC, ACC, insula, and other cortical regions [22, 23, 46, 100]. Thus, chronic smoking in PSU may be associated with different effects on cortical morphometry than in ALC or drug-free controls. Reasons for the observed *larger* prefrontal volumes in smoking vs. non-smoking PSU are uncertain (and may be related to the rather modest sample size). Cortical hypertrophy may be related to potentially long-lasting inflammatory processes from the interaction of smoking and past polysubstance use [101]. Alternatively, thicker regional cortices may be associated with greater cognitive demand on these brain regions, which are important for higher cognitive functions, such as behavioral control, particularly in the face of negative affect and stress (e.g., [102]). As the larger morphometric measures in smoking vs. non-smoking PSU relate to better cognitive test performance, the latter interpretation appears more likely. However, further studies in a larger cohort are necessary to replicate the findings and refine their interpretation.

### Structure-function relationships

#### Neurocognition

The nature and pattern of correlations between regional cortical morphometry and cognition were distinctly different between the three groups. In PSU, smaller DPFC and ACC volumes and surface areas were related to worse performance in processing speed and cognitive efficiency (left ACC volume and surface area only). These correlations were not exhibited in ALC and CON. Many moderately strong correlations of morphometry to cognition were observed in ALC but not in PSU, with working memory being the most distinct. Namely, smaller volumes and surface areas in the DPFC, ACC, OFC and insula (bilateral left only) of ALC correlated to worse working memory, whereas PSU failed to show any relationships between morphology and working memory. Identifying distinct areas of deficit or sensitivity for different substance using populations is important as it can guide the development of specific treatment interventions, such as cognitive remediation [103].

Cortical thinness has been associated with reduced attention, judgment and decision making in cocaine users [87]. Surprisingly, thinner cortices in the right ACC of PSU showed moderate associations with better executive functioning. Thicker right ACC of PSU may reflect stimulant associated neuroinflammation [101], and behaviorally, poorer executive functioning. Additionally, right ACC may be a particularly salient brain region affected in PSU, and although not different from controls, the 1-month-abstinent PSU may show dysmorphology earlier in abstinence, similar to findings in ALC at one week of abstinence [90]. However, these structure-function relationships should be interpreted with caution as they were exploratory. Nonetheless, PSU appear unique in morphology related cognitive dysfunction when compared to ALC and CON.

#### Inhibitory control

It appears that prefrontal morphometric abnormalities in PSU are at least in part related to impulsivity and risk-taking. Correlations with self-reported impulsivity (BIS) were generally few and moderate within all groups. Specifically, increased impulsivity (higher BIS scores) was related to thinner DPFC cortices in both PSU and CON, but not in ALC. Further, in PSU but not in ALC or CON, greater risk-taking (BART adjusted average pumps) was associated with larger volumes and surface areas of the DPFC and ACC. Since smoking PSU show the larger of these morphometric measures, smokers may drive this correlation by taking greater risks than non-smoking PSU at this task. Finally, only in CON but not in ALC or PSU was a measure of decision-making (IGT) negatively related to DPFC and ACC morphometrics.

In previous reports, higher measures of impulsivity correlated positively with left inferior and medial superior frontal clusters (DPFC regions) and negatively with insula volume in 5-week-abstinent cocaine users [104], and smaller OFC volume in multi-year abstinent PSU was associated with greater persistence to play “bad” cards, a modified gambling task measure [96]. In contrast to our measures of cognition in which the pattern of correlations of volumes and surface areas of the DPFC to cognitive test performance was similar in PSU and ALC, our measures of inhibitory control (BART/IGT/BIS-11) did not show overlapping patterns.

Taken together, some structure-function relationships were distinctly different between the substance dependent groups, indicating that the nature of the substance dependence, that is alcohol vs. polysubstance dependence, appears to alter these normal relationships differentially. Because of these different structural alterations and their functional ramifications, treatment approaches that are tailored to the specific deficits and morphometric correlates of the different substance dependent populations may increase the efficacy of current substance dependence treatment.

### Effects of substance use quantity/frequency and abstinence duration on brain morphometry

Longer duration of stimulant use has been associated with reduced volumes in the frontal cortex and limbic system [105] (note, however, that these relationships were not corrected for age). Here, we did not detect any correlations between abnormal morphometry and substance use quantity, age of onset of heavy drinking, or smoking severity. Thus, the unique abnormal morphometry observed in this report appears to be related more to substance use status than amount of substances used.

The PSU in this study were abstinent from substances (both alcohol and illicit drugs) for an average of about one month; we have no structural data in PSU abstinent for less than one month. However, previous 1.5T morphometric data from 1-week-abstinent ALC indicated that, while gray matter volume from the entire frontal lobe was not significantly smaller than in non-alcoholic CON [29, 34], gray matter volume in the DPFC was reduced [46], and cortices in the DPFC, insula, OFC, and entire frontal lobe were thinner than in CON [32]. Assuming volumetric recovery with abstinence from alcohol, any initial volume reductions may have largely normalized in the 1-month abstinent ALC examined in this morphometric study at 4T (but see [36]). Similarly, cortical gray matter volume loss may be more apparent/widespread in currently using or 1-week-abstinent PSU, but cortical atrophy may not have fully recovered at one month of abstinence, at least in the left OFC and right DPFC shown in this study (alternatively, the structural abnormalities may be premorbid). Previous studies in substance dependent individuals, who report cocaine as their primary drug of choice but also use other substances, reported smaller volumes of OFC, right ACC, and right insula at five weeks of abstinence [104], lower gray matter densities in ACC and OFC at three weeks of abstinence [94], and smaller and thinner insula and DPFC [106] as well as smaller OFC and insula in current users [92, 93]. Persistent OFC volume loss has been described in individuals abstinent from cocaine, alcohol, methamphetamines and cannabis for several years [96]. Thus, the regional prefrontal volume reductions in our 1-month-abstinent PSU are not inconsistent with previous reports and they also likely reflect partial recovery from prefrontal and insular gray matter dysmorphometry during abstinence from substances.

## Conclusions

In sum, individuals with comorbid alcohol and stimulant use disorders (PSU) at one month of abstinence have normal cortical thickness throughout anterior brain but significantly smaller volumes of OFC and trends to smaller DPFC than drug-free controls. Their morphometric alterations are distinctly different from those of “pure” alcohol dependent treatment seekers at one month of abstinence, despite similar age, drinking and smoking histories. PSU also showed distinct neurocognitive associations to regional morphometrics when compared to alcohol use disorder-only treatment seekers and drug-free light drinking controls. These group differences are presumably associated with the diagnosis of multiple substance use disorders rather than amount of use, and underlie different cognitive and behavioral correlates. Additionally, PSU showed abnormal morphology related to cigarette smoking. However, these differences were not in the postulated direction, thus future studies should seek to replicate these findings and illuminate potential mechanisms of these truly unique differences. Taken together, PSU may require different pharmacological and/or behavioral interventions than those provided to “mono-substance” users. Given our limited knowledge about brain changes in this large understudied population of polysubstance users, the difficulties in treating this complex population, as well as the results of this study, large longitudinal investigations are needed to assess more definitively the unique differences in regional morphometrics, their related functionality, and potential injury mechanisms across different substance dependent groups. Such studies may guide urgently needed and better-targeted pharmacotherapy and behavioral treatments for different substance using populations.
